# Editorial: Antimicrobial Use, Antimicrobial Resistance, and the Microbiome in Food Animals

**DOI:** 10.3389/fvets.2020.638781

**Published:** 2021-01-18

**Authors:** Moussa S. Diarra, Xin Zhao, Patrick Butaye

**Affiliations:** ^1^Guelph Research and Development Center, Agriculture and Agri-Food Canada, Ottawa, ON, Canada; ^2^Department of Animal Science, McGill University, Montreal, QC, Canada; ^3^Ross University, School of Veterinary Medicine, Basseterre, St Kitts and Nevis; ^4^Department of Pathology, Bacteriology and Avian Diseases, School of Veterinary Medicine, Ghent University, Merelbeke, Belgium

**Keywords:** food animal, antimicrobial resistance, antimicrobial use, microbiota, microbiome

## AMU and AMR

Despite the growing demand of animal protein, livestock, and poultry productions around the world are facing constraints including their consequences on environment, food safety, and animal health and welfare. No doubt that antibiotics significantly contributed to the increasing productivity of food-animals. Sir Alexander Fleming, who discovered the first antibiotic, was also the first person to warn about antimicrobial resistance (AMR) during his Nobel price speech in 1945. Nevertheless, nothing happened at that time. One of the first reports to seriously criticize our unnecessary and improper antimicrobial use (AMU) that led to some partial restrictions, was the Swann's report, released in the late 1960s. However, this was only dealing with the AMU as growth promoters and led only to partial restrictions: only non-therapeutically used antimicrobials could be used as growth promoters. It was only as late as in the late 1990s that a real movement started with the pressure for a total ban on the use of antimicrobial growth promotors which was adopted in the early 2000s, but only in Europe (1). Nevertheless, this was the start on our rethinking about AMU, and the first question was about the amount of antimicrobials used in both humans and animals. Indeed, we did not even know this. Articles in this Research Topic handled that subject (Gemeda et al.; Lardé et al.). This knowledge leads to a better understanding of the selection pressures and allowed to concisely make decisions on potential reductions, which came later in several countries. Of course, one should be aware that antimicrobials can have positive effects on certain syndromes and adverse effects of reducing AMU should be investigated so they can be counteracted by alternatives. One of these potential adverse effects was described by Davedow et al. however, they indicate possibility to reduce the tylosin use in feedlot cattle without impacting animal productivity.

## Microbiota and Microbiome

The digestive tract is one of the largest immune organs responsible for a large proportion of immune responses, so optimal gut health in production animal is vital for their growth and performance. Gut health, which is linked to microbial community, can be achieved through a combination of nutrition, microbiology, immunology, and physiology approaches. When gut health is compromised, digestion, and nutrient absorption are affected which, in turn, can lead to a greater susceptibility to diseases that ultimately lead to an increase of antibiotics for treatments. It is also well-known that antimicrobials may have direct effects on the microbial communities. This has been investigated frequently on the selection of antimicrobial resistant bacteria but using mainly culture dependent methods. With the advent of the new high throughput sequencing methods, we can now have a better view on what happens in diverse ecosystems of the body. Turcotte et al., showed that short term effects are not to be expected on AMR, but several years are necessary. Meanwhile, alterations in the microbiome were also noted. Contrary, in the study on the nasopharyngeal microbiome and the effects of tilmycosin, minimal changes in the microbiome were detected (Zeineldin et al.). The selective effect and alteration of the microbiomes of antimicrobials is also influenced by the way the antimicrobial is administered, and likewise the reduction of resistance may be obtained (Ricker et al.) As such reduction and alternative applications of antimicrobials may also aid to breakdown resistance, albeit only at the long term. Perseverance is necessary. Nevertheless, more studies are needed to fully understand the total effects of antimicrobials on the selection of resistant bacterial, animal performance, and health as well as microbial communities and microbiome including the resistome.

## Alternatives and AMR

The clinical AMU will remain and a reduction in AMR, when reducing/eliminating unnecessary use, is taking time, but it works as shown in The Netherlands (2). Taking this into account, there is an urgent need for alternatives to antimicrobials and several strategies are under investigation. The road to alternatives is not paved smoothly and this was exemplified in the article of Kurt et al.. Apart from real agents killing bacteria, like phages, indirect strategies are also possible as exemplified in this Research Topic by Alizadeh et al., where immune stimulation was demonstrated by in ovo application of probiotic bacteria. Plant based alternatives were also handled and were found to improve performance, liver immunity, and intestinal health of broiler chicken (Das et al.). Not only plant-based products but also organic acid as formic acid may help in reducing the AMU and have been shown to reduce the prevalence of non-typhoid *Salmonella* (Ricke et al.). Non-typhoid *Salmonella* is still a major foodborne agent, frequently resistant to antimicrobials. Specifically, for *Salmonella*, several serotypes are resistant to multiple drugs, while others, are way more susceptible, though they are in the same ecosystem (Gu et al.). This is also an interesting finding as it may identify bacterial factors that inhibit the acquisition of resistance genes. Reduction of AMU can also be improved by better diagnostics, excluding viral infections leads to a lesser use of antibiotics as they have no effect on viruses. Isothermal tests described in this Research Topic seems allowing an easy, fast and on the field diagnosis of bacterial pathogens (Conrad et al.).

## Perpectives

It is clear that antimicrobial resistance is a complex problem for which multiple solutions need to be applied, though effects are only to be expected at a long term. Several pathways can be taken and some of them were presented in this Research Topic, though many others are under investigation. It is clear that we also should take into account of the microbiomes and more studies in this field are necessary to understand its roles and functions knowing that it is in part also shaped by bacteriophages. The role of the latter is in general a bit neglected, though interest is increasing, not only for their role in shaping the microbiomes but also as therapeutics. Furthermore, understanding how feeding programs including organic farming impact the beneficial microbes, pathogens, and AMR, will help guide dietary or management practices. Given the AMR complexity mentioned above, tackling this issue requires an “One Health” approach, which is based on the principle that human and animal health are interconnected in relation with a heathy environment. Efforts in one will not be sufficient to cover all current problems with AMR but also in the human and environmental ecosystems, measures should be taken to reduce its burden. The existence of high abundance of various antibiotic resistance genes (ARGs) in animal manure and subsequent environmental contamination may be avoided by treatment approaches, such as composting (thermophilic composting and vermicomposting) and anaerobic digestion.

## Author Contributions

All authors listed have made a substantial, direct and intellectual contribution to the work, and approved it for publication.

## Conflict of Interest

The authors declare that the research was conducted in the absence of any commercial or financial relationships that could be construed as a potential conflict of interest.
